# Copper (II) Level in Musts Affects Acetaldehyde Concentration, Phenolic Composition, and Chromatic Characteristics of Red and White Wines

**DOI:** 10.3390/molecules29122907

**Published:** 2024-06-19

**Authors:** Francesco Errichiello, Luigi Picariello, Martino Forino, Giuseppe Blaiotta, Ernesto Petruzziello, Luigi Moio, Angelita Gambuti

**Affiliations:** Department of Agricultural Sciences, Grape and Wine Science Division, University of Naples “Federico II”, 83100 Avellino, Italy; francesco.errichiello@unina.it (F.E.); luigi.picariello@unina.it (L.P.); blaiotta@unina.it (G.B.); ernesto.petruzziello@unina.it (E.P.); moio@unina.it (L.M.); angelita.gambuti@unina.it (A.G.)

**Keywords:** copper (II), copper (II) in musts, copper (II) in wine, organic farming, acetaldehyde, polyphenols, polymeric pigments, wine chromatic characteristics

## Abstract

Copper (II), a vital fungicide in organic viticulture, also acts as a wine oxidation catalyst. However, limited data are currently available on the impact that maximum allowed copper (II) ion doses in wine grapes at harvest can have on aged wine quality. This was the focus of the present study. We investigated the copper (II) effects by producing both white and red wines from musts containing three initial metal concentrations according to the limits set for organic farming. In detail, the influence of copper (II) on fermentation evolution, chromatic characteristics, and phenolic compounds was evaluated. Interestingly, the white wine obtained with the highest permitted copper (II) dose initially exceeded the concentration of 1.0 mg/L at fermentation completion. However, after one year of storage, the copper (II) content fell below 0.2 ± 0.01 mg/L. Conversely, red wines showed copper (II) levels below 1.0 mg/L at the end of fermentation, but the initial copper (II) level in musts significantly affected total native anthocyanins, color intensity, hue, and acetaldehyde concentration. After 12-month aging, significant differences were observed in polymeric pigments, thus suggesting a potential long-term effect of copper (II) on red wine color stability.

## 1. Introduction

Copper (II) is one of the most extensively used pesticides in agriculture, in particular for organic products. It is also a micronutrient for plants, but, if absorbed in too high quantities, it can be toxic. For this reason, its use is quite controversial due to environmental risks, as it is a slightly mobile element that tends to accumulate in the surface layers of the soils [1]. Copper (II) is a heavy metal that acts as a catalyst for many chemical reactions. In plant cells, it takes part in photosynthetic electron transport, and it is an integral part of enzymes such as polyphenol oxidase, monoamine oxidase, and other phenolases. However, organisms are usually unable to fully control copper (II) entrance into their cellular structures. They also lack the capability of fully neutralizing its dangerous effects when excessive accumulation in cells and tissues occurs. Organisms thriving in an environment with elevated amounts of copper (II) are subjected to higher risks of suffering from several developmental and reproductive disorders [2].

Copper (II) has been used in viticulture for more than 150 years, at concentrations of up to 80 kg/ha per year. This has led to the accumulation of copper (II) in the topsoil of many vineyards [3]. Adverse effects of copper (II) have also been noted in wine production. Apart from health concerns about the risk associated with drinking wines contaminated by excessive levels of copper (II) [4], several enological issues in musts and wines seem to be clearly entwined with copper (II).

Despite its unfavorable ecotoxicity [5], the use of copper (II) is still accepted due to its unique properties as a wide-spectrum fungicide and bactericide. However, a recent re-evaluation of copper (II) in the European Union (EU) has further restricted its use. Consequently, the future of viticulture appears to be currently dependent on the availability of copper (II) unless alternatives are identified. Additionally, always due to copper (II)’s negative environmental impacts, the European Commission has included cupric plant protection products among the substances to be replaced [6].

Concerning the transport mechanism of copper (II) across the soil–plant–fruit–wine system, reports in the literature are still quite poor. In a study conducted in 2019 [7], it was observed that plants are able to limit the passage of copper (II) across their compartments. The roots keep most of the metal in their structures as a protective mechanism against toxicity, thus reducing absorption (soil–root) and translocation to the sprout (root–sprout). Furthermore, in another study, it was observed that copper (II) competes with zinc for the same binding sites in the soil where copper (II) is preferentially adsorbed [8].

When grapes are harvested, part of copper (II) (both from the surface of the grapes and absorbed from the soil) is transferred to musts and, successively, to wines [7]. Another important issue is the amount of metal transfer from grapes to finished wines. Once transferred, the copper (II) effects in winemaking depend on the concentration. In controlled (up to 0.1 mM, 6.35 mg/L) doses, copper (II) can be used by yeasts as a survival element [9,10]. Also, it can be useful to remove undesirable reductive aromas that may arise during fermentation [11]. However, levels higher than 20 mg/L disrupt yeast activity, potentially leading to sluggish or even stuck fermentation [12]. This, in turn, can cause elevated hydrogen sulfide (H_2_S) concentrations in the finished wine, contributing to unpleasant aromas [13]. The problems associated with copper (II) extend beyond fermentation. Residual copper (II) in wine has been linked to both oxidative and reductive spoilage processes: oxidative reactions catalyzed by copper (II) and involving phenolic compounds can be very dangerous for wine quality determining the production of hydrogen peroxide, quinones, and acetaldehyde [14]. Concerning the reductive off-flavor, Clark et al. [11] shed some light on the negative interaction between residual copper (II) and hydrogen sulfide in wines stored under low-oxygen conditions. This interaction can lead to the formation of further undesirable aromas, highlighting the complexities associated with copper (II) management. Additionally, copper (II) can destabilize wine proteins, leading to the formation of haze, a phenomenon known as “copper (II) casse” [15].

Given these potential drawbacks, the meticulous control of copper (II) is necessary throughout the winemaking process. The EU regulations suggest various strategies to minimize copper (II) exposure, whose use as a fungicide is currently restricted in Europe [16]. In detail, its use in organic farming is limited to 28 kg/ha over a seven-year period, with a maximum of 6 kg/ha per year. Moreover, the detection limit on wine grapes at harvest is set at 20 mg/kg [17]. Despite the EU regulations, to the best of our knowledge, few studies have evaluated the effect of admitted copper (II) levels in musts on wine phenolic composition and chromatic characteristics as well as on the evolution of wines. Sun et al. [18] evaluated the effects of different concentrations of copper (II) in grape must (from 0 to 96 mg/L) on the phenolic compound contents and chromatic characteristics of Cabernet Sauvignon, while the influence of juice metal concentrations and bentonite fining on the copper (II) speciation profile of Chardonnay wine was examined by Rousseva et al. [19] for a maximum value of added copper (II) (as copper (II) (II) sulfate pentahydrate) in juice of 5.8 mg/L. This study demonstrated that metals originating from the juice are able to impact the rate of oxygen consumption in the final wine. This was particularly significant for copper (II). Its total, residual, and cationic forms in wine were better correlated with oxygen consumption in comparison with iron.

Given the increase in organic crops and the lack of information on the effect of copper (II) on the evolution of phenolic compounds in wines over aging, the aim of this work was to evaluate the effect of two levels of copper (II) in musts (10.0 and 20.0 mg/L, added as copper (II) sulfate pentahydrate CuSO_4_ • 5H_2_O) on the fermentation evolution, acetaldehyde concentration, chromatic characteristics, and phenolic compounds of both white and red wines by assuming that a complete transfer of copper (II) occurs from grape to must. To this aim, we selected two varieties, namely the red one Aglianico (*Vitis vinifera* L.) and the white one Greco di Tufo (*Vitis vinifera* L.). These two cultivars are among the main wine grapes cultivated in Campania, Italy, for the production of well-known DOCG (Denominazione di Origine Controllata e Garantita) wines.

## 2. Results and Discussions

Two grapevine varieties, a red (Aglianico, AG) and a white (Greco di Tufo, GR) one, were used to investigate the effect of adding different copper (II) levels (high: 20 mg/L, low: 10 mg/L) to musts before fermentation. In order to avoid possible copper (II) residues on grape surfaces coming from previous in-field treatments, harvested grapes were accurately washed and dried before crushing. This procedure did not remove possible copper (II) naturally occurring inside the berry and likely depending upon the soil condition. The effects on fermentation progress, acetaldehyde content, color characteristics, vanillin-reactive flavans (VRF), BSA-precipitable tannins, polymeric pigments, and total phenols were evaluated during fermentation, at the end of alcoholic fermentation, and after 12 months of aging.

### 2.1. Fermentation Evolutions and Copper (II) Evolution during Fermentations

A comparison of the fermentation evolutions of red and white wines is shown in Supplementary Figures S1–S3.

The alcoholic fermentation process differed in duration between the red and white grapes. As shown in Supplementary Figure S1A, the red Aglianico must completed fermentation in 19 days, while the white Greco di Tufo must required a longer fermentation period up to 34 days (Supplementary Figure S1B). This difference is likely attributable to the varying nitrogen content in the musts. Red grape must, containing both skin and seeds in addition to pulp, usually has a higher concentration of nitrogen sources compared to white must, which typically consists of pulp alone [20]. Since nitrogen plays a crucial role in yeast metabolism during fermentation, the lower nitrogen content in the white grape must likely delayed the onset of the exponential fermentation phase, where yeast growth and sugar conversion rapidly increase.

During alcoholic fermentation, the copper content was monitored, and a comparison between the samples is shown in Table 1 and Table 2. It is important to underline that time zero does not represent the baseline for copper (II) content evaluation, as the copper (II) analyses were carried out approximately four hours after sampling, as detailed in Materials and Methods. This delay may have resulted in an apparently non-cumulative effect, since some of the copper (II) may have started precipitating and/or being absorbed by yeasts before the analyses [12,21,22]. The analysis at time zero for red grape Aglianico (Table 1) showed an average copper (II) concentration of 5.35 ± 0.64 mg/L. These concentrations are lower than those reported in the literature by Fregoni and Corallo [23], where the total copper (II) residue concentration in grapes ranged from 10 to 56 mg/L. As in our study, grapes were washed before winemaking to eliminate each possible residual copper (II), this first datum confirms the efficacy of the washing step carried out on grapes before crashing especially if copper (II) is used in vineyards. The levels found at zero time for AG10 and AG20 are consistent with the amount of copper (II) added at the beginning of the experiment. As expected, significant differences (*p* > 0.05) were found between control and treated wines at time zero as well as at day 1, but no significant differences were found between control and treated samples from day 5 to the end of alcoholic fermentation. Our results showed a range of residual copper (II) at the EAF from 0 to 1 mg/L, in agreement with results previously observed for Cabernet Sauvignon [18]. These results highlighted that, even if there are high amounts of copper (II) in red grapes, after alcoholic fermentation, the copper (II) values fall below the threshold limit of 1 mg/L, in agreement also with current EU regulation for wines [24].

The same behavior during fermentation was observed for the white Greco di Tufo variety (Table 2). At time zero, the average copper (II) concentration in control wine was 3.94 ± 0.27 mg/L. Unlike the Aglianico variety, we could observe (Table 2) a significant difference between treated and control samples throughout the alcoholic fermentation with a constant higher content of copper (II) in GR20 and GR10 with respect to the control samples. This trend was not observed for the red variety, which differs from the white one by the presence of pomace during fermentation. Probably, the pomace induces mineral adsorption phenomena by subtracting copper (II) from the aqueous medium [25]. At the end of alcoholic fermentation (EAF), unlike Aglianico samples, GR10 and GR20 showed the highest residual amount of copper (II), exceeding the EU legal threshold limit value of 1.0 mg/L (until 1.66 ± 0.04). For both fermentations, a greater loss of copper (II) was detected in the last phases of fermentation, probably because in these steps, the yeast cells acted as adsorbent material for this element. Previous researchers also showed that during fermentation, a large proportion of copper (II) is precipitated with yeast cells [22,26,27]. These results are of great interest because the control of the copper (II) level is of critical importance for wine, because copper (II) acts as catalyst of chemical oxidation reactions, and its catalyst activity in wines is dependent upon concentrations, as shown by Cacho et al. [28] in an experiment in which they evaluated the levels of copper (II) ranging from 0.58 to 8.95 mg/L. Concerning microbial evolution during the fermentation of Greco di Tufo and Aglianico grapes (Supplementary Figure S3), a significant inhibitory effect of copper (II) on non-*Saccharomyces* yeasts was observed for Greco grapes. This effect was proportional to the amount of copper (II) added. Albeit not relevant to our experiment, in which selected yeasts were used, these findings could either have interesting implications for non-inoculated musts or for fermentation in which non- *Saccharomyces* yeasts are used. As a consequence of the copper (II) effect, *Saccharomyces* yeasts may gain a greater competitive advantage over non-*Saccharomyces* ones in all fermentations, potentially establishing dominance over a shorter period of time.

### 2.2. Acetaldehyde Production during Fermentations

In this study, the monitoring of acetaldehyde was carried out with a double purpose: aldehyde is an important secondary metabolite from microbial activity, and it is also the main product of the chemical oxidation of wine [29].

**Figure 1:**
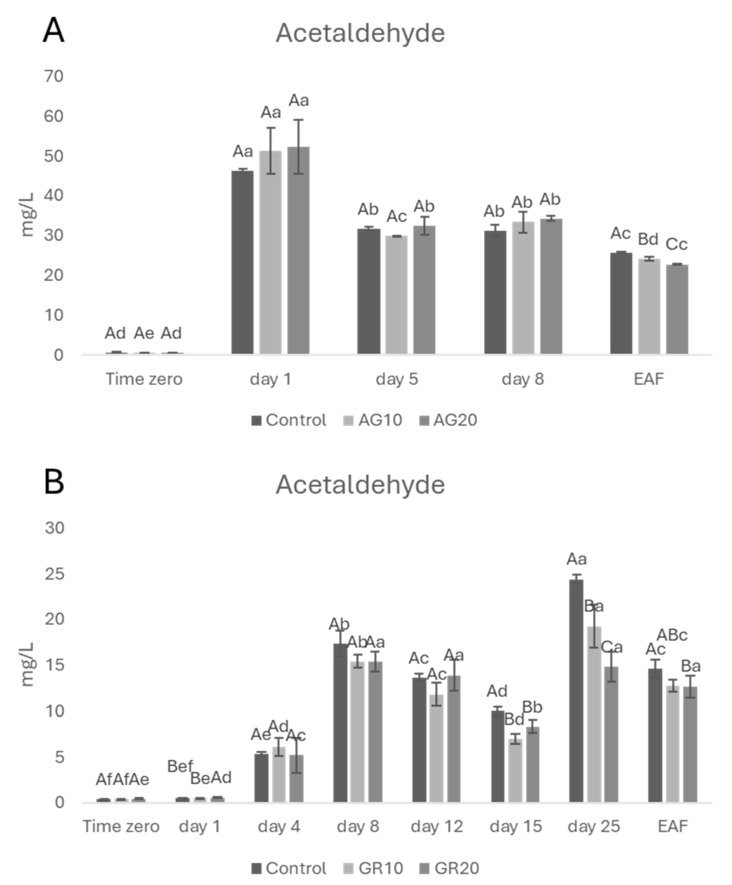
The concentration of acetaldehyde (**A**) Aglianico, (**B**) Greco di Tufo during alcoholic fermentation. Data are presented as the mean ± standard deviation (SD) across four replicates. Letters (A, B) indicate statistically significant differences between treated wines at the same time point. Letters (a, b, c, d, e, f) indicate significant differences for a single treated group over different time points (*p* < 0.05).

Analyses of acetaldehyde were carried out during wine fermentation. In red wines (Figure 1A), acetaldehyde increased (day 1) and then decreased (day 5) until the end of alcoholic fermentation. This trend is well known and common to all fermentations: acetaldehyde increases during the first days of fermentation [30] and successively decreases due to its reduction to ethanol by NADH [31] as well as to its involvement in reactions with wine components including phenolic compounds [32,33]. In regard to the possible effect of copper (II), previous research suggests that higher copper (II) concentrations can stress yeasts, leading to increased acetaldehyde production [34]. Therefore, we would have expected the treated wines (AG20 having the highest copper (II)) to have the highest concentrations of acetaldehyde, but no statistical differences between treated and untreated wines were detected during the first phases of the fermentation process. This is probably due to the low level of copper (II) in treated wines with respect to the study of Liang and Zhou [34]. At the EAF, however, the control wine showed a higher content of acetaldehyde compared to the treated samples (AG10 and AG20). This finding is quite surprising, but it can be explained by taking into account the higher reactivity of acetaldehyde towards phenolic compounds [35]. In addition, when the role of acetaldehyde in phenolic compound solubilization and precipitation during winemaking was evaluated, Teng et al. [36] demonstrated that the acetaldehyde-mediated pathway predominated in pigment formation, thus suggesting that all conditions during winemaking which give rise to acetaldehyde appear to be important factors involved in the formation of stable, soluble non-bleachable pigments. Since all these reactions occurred during wine oxidation, well-known evidence that copper (II) and iron accelerate the reaction with oxygen [37] explains the observed trend for copper (II)-treated wines.

Regarding the Greco di Tufo wines, during the first fermentation phase (Figure 1B), all the experimental samples did not show any statistical difference in terms of acetaldehyde content up to day 15. However, starting from day 15, fermentations appeared to struggle, and the rate of sugar consumption quickly slowed down (Supplementary Figure S1). This suggests that the yeasts underwent stress around that time. Several factors could have contributed to this stress response. After day 15, the absence of oxygen and essential survival factors for yeasts [38], combined with the increasing ethanol concentration in the fermenting must [39], likely limited yeast viability and slowed alcoholic fermentation. It is important to note that this effect is more pronounced in white grape musts compared to red ones. The presence of skins and pulps in red wines provides yeasts with additional nutrients and precursors for essential survival factors, potentially delaying or mitigating the stress response observed in white grape fermentations. This slowdown could explain the observed higher acetaldehyde content in control wine with respect to GR10 and GR20.

At the EAF, the control sample still showed a slightly but significantly higher acetaldehyde content compared to the GR20 treatment, probably because copper (II) may have a positive effect on yeast metabolism [40]. Further investigation is needed to elucidate the observed results.

### 2.3. Effect of Copper (II) on Phenolic Composition and Chromatic Characteristics of Red and White Wine during Fermentation

To understand the impact of copper (II) on the red wine fermentation process, color intensity (CI), hue, monomeric native anthocyanins (Supplementary Table S4), total native anthocyanins, and polymeric pigments were evaluated through HPLC and spectrophotometric analysis, as shown in Table 3.

During the fermentation process, the evolution of CI and of polymeric pigments was monitored in all samples (Table 3). A general increase in CI was observed over time. This is due to the release of phenolic compounds by the skins and seeds, especially anthocyanins absorbing at 520 nm, and to the chemical reactions leading to the formation of polymeric pigments [33]. These latter are formed during maceration and wine aging [41] thanks to the reactions of condensation between flavan-3-ols (including catechin, epicatechin, proanthocyanidins) and anthocyanins either mediated or not by acetaldehyde [33,42]. At the EAF, significant differences among samples were detected. A decrease in color intensity and the total native anthocyanins with an increase in the hue was observed in samples with major amounts of copper (II) (AG20). Probably, the effect of copper (II) as a catalyst for oxidation reactions led to the loss of these compounds. This behavior was not seen in sample AG10. Interestingly, the analysis of polymeric pigments at the EAF revealed a statistically significant difference in the sample with the most copper (II) (AG20) compared to the control. The lower acetaldehyde content in the AG20 sample (Figure 1A) might have played a role in influencing the overall color component profile, potentially affecting the formation or stability of certain pigments besides the observed increase in polymeric pigments. Ćurko et al. [43] found comparable outcomes regarding anthocyanins in the initial months of aging following the micro-oxygenation of wines with added metals, which supports the evidence that metals would promote increased ethyl-bridged polymerization reactions, as suggested by Danilewicz [44].

**Figure 2:**
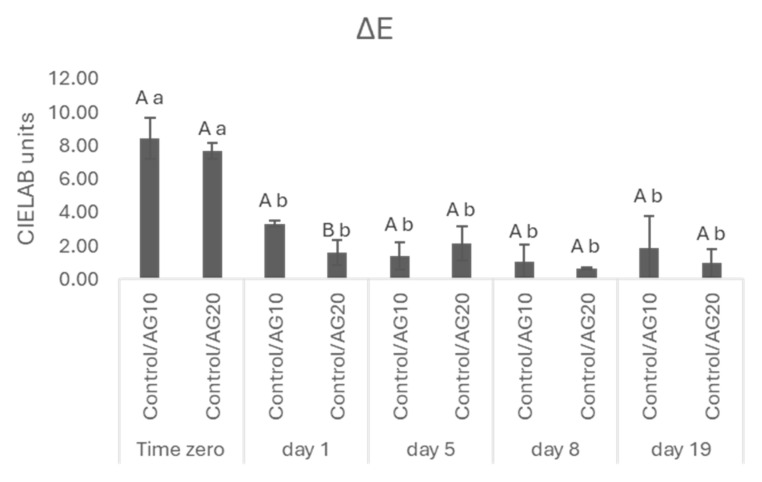
ΔE = [(ΔL*)2 + (Δa*)2 + (Δb*)2]1/2 in CIELAB units. Data are presented as the mean ± standard deviation (SD) across four replicates. Letters (A, B) indicate statistically significant differences between treated wines at the same time point. Letters (a, b) indicate significant differences for a single treated group over different time points (*p* < 0.05).

The analysis of chromatic characteristics was completed by the determination of ΔE which allows us to measure the color perception by human eyes (Figure 2). ΔE represents the color variation between the control and treated wines, and values greater than two (>2) CIELab units indicate that wines show a difference detectable to the human eye [45]. ΔE between the control and each sample was higher than 2 only at the beginning of AF. In the last phases of AF, we observed a decrease in ΔE to values lower than 2.

During AF, all the parameters related to phenolics increased until the EAF (Table 4). Three different groups of phenolic substances were determined by evaluating their specific reactivity: (i) Vanillin-reactive flavans (VRFs); this parameter is basically correlated with low-molecular mass proanthocyanidins, including dimers, trimers, and tetramers of flavan-3-ols that contribute to wine bitterness [46,47]; (ii) BSA–tannin interactions involve high-molecular weight proanthocyanidins. These proanthocyanidins can be precipitated by BSA, and the degree of precipitation increases with increasing polymerization (or size) from trimers to octamers. This suggests that any changes in tannin composition and size can affect their ability to bind with BSA [48]. Finally, in order to have an overview of the phenolic fraction, we also evaluated (iii) the total phenolic fraction by resorting to the reaction with iron. In the AG20 wines, VRFs and BSA-reactive tannins were significantly lower than in both AG10 and the control. In regard to the total phenol amount, a clear trend as a function of the copper (II) amount was not detected. This could be due to the fact that phenols can undergo hydrolytic cleavage in the presence of oxygen with consequential formations of new intra- and inter-molecular bonds that can alter the reactivity towards the reagents used for their determination [49,50].

Regarding the Greco di Tufo wine, a decrease in HCAs was observed during alcoholic fermentation in all samples (Table 5). HCAs are the major phenols in grape juice and the major class of phenolics in white must. These compounds are also the first to be oxidized by polyphenol oxidase during the first phase of winemaking, and, subsequently, they initiate browning reactions. These reactions can be highly deleterious for the color of white wine [51]. A parameter that can be correlated to the phenolic compounds and oxidation of white wines is the absorbance at 420 nm (Abs420 nm). This is considered an indicator of the degree of browning of musts and white wines during alcoholic fermentation and storage [52]. The lowest value in Abs 420 nm was registered in all the treated and untreated samples at the end of alcoholic fermentation. This trend may be related to the possible polymerization and precipitation of quinones leading to brown pigments [53], as well as their potential absorption by the yeast cell wall [54]. At the EAF, GR20 showed a higher amount of these compounds. Probably, the higher content of copper (II) induced a higher level of the oxidative process and hence an increased production of xanthylium cation pigments [55,56]. Regarding the total phenol content, no significant differences were observed between zero time and the EAF.

### 2.4. Effect of Copper (II) on One-Year Aged Wines

After 12 months of aging, all samples were analyzed in order to quantify the residual copper (II) in wine and lees as well as to identify changes affected by copper (II) on acetaldehyde, total phenols, BSA-reactive tannins, and vanillin-reactive flavans.

Regardless of the initial copper (II) content, after 12 months of aging, the content of copper (II) is below the EU threshold limit of 1 mg/L (from 0.17 mg/L to 0.20 mg/L for Greco and from 0.01 mg/L to 0.06 mg/L for Aglianico). No significant differences among treatments were observed. The only difference between the white and red samples was the copper (II) content. Aglianico showed a lower copper (II) content compared to Greco di Tufo. Of particular interest is the amount of copper (II) found in the lees: the amount was different for white and red wines. It ranged from 124 mg/kg to 182 mg/kg for Greco and from 226 mg/kg and 361 mg/kg for Aglianico. Probably, the higher content of lees in red wines and the presence of more solid residues in them are responsible for the higher level of copper (II). Our data are consistent with a review concerning the possible re-use of wine lees, which reported a wide range for the copper (II) content in wine lees (13–1187 mg/kg) [57]. It is therefore important to consider the content of this metal in lees, especially when used in extraction protocols to extract valuable components from them.

**Figure 3:**
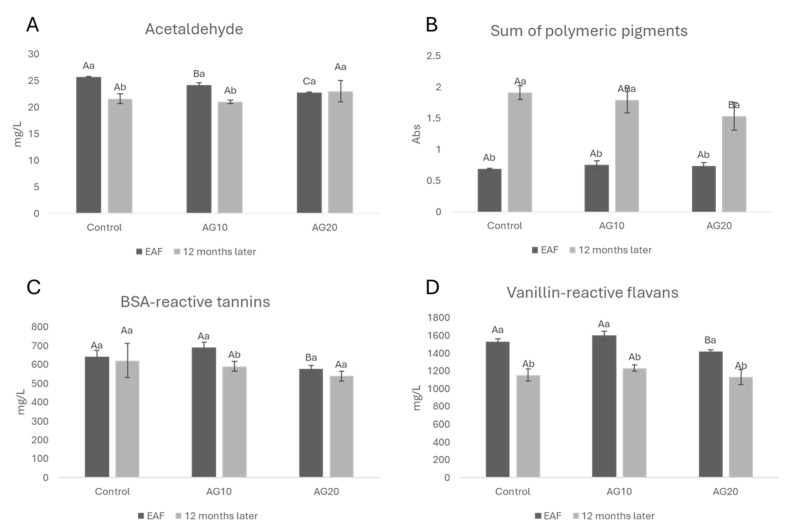
Acetaldehyde (**A**), the sum of polymeric pigments (**B**), BSA-reactive tannins (**C**), vanillin-reactive flavans (**D**) at the end of alcoholic fermentation (EAF) and after 12 months of aging in red Aglianico wines. Data are presented as the mean ± standard deviation (SD) across four replicates. Letters (A, B) indicate statistically significant differences between treated wines at the same time point. Letters (a, b) indicate significant differences for a single treated group over different time points (*p* < 0.05).

In Figure 3, the concentration of acetaldehyde (Figure 3A), the sum of polymeric pigments (Figure 3B), BSA-reactive tannins (Figure 3C), and vanillin-reactive flavans (Figure 3D) at the EAF and after 12 months of aging are shown. The decline in acetaldehyde, which possesses an electrophilic carbonyl group, is due to its numerous reactions with a number of nucleophiles naturally occurring in wine, including alcohols, thiols, sulfur dioxide (SO_2_), and flavonoids [32]. Regarding polyphenols, acetaldehyde can react with anthocyanins and flavanols to form methylmethine-bridged compounds [58], which can react with additional acetaldehyde, anthocyanins, and flavanols to generate polymeric-type structures that can alter wine sensory attributes [59]. Additionally, the reaction between anthocyanins and acetaldehyde can lead to the formation of stable pyranoanthocyanins [60]. In Figure 3B, the concomitant increase in polymeric pigments can be easily correlated to acetaldehyde, VRF, and BSA-reactive tannin loss. VRFs, which are mainly proanthocyanidins with low molecular masses, Ref. [61], decreased in all of the samples after 12 months of aging.

After 12 months of aging, the loss of acetaldehyde was detected in all samples except AG20 with respect to the EAF (Figure 3A). This could be ascribed to the presence of copper (II), as shown by Kontoudakis et al. [62] who demonstrated that oxidative storage increases the most oxidative catalytic form of the metal, and as a consequence, the production of acetaldehyde and its further reactions during wine aging should be favored. This possible explanation seems to be in apparent contrast with the fact that the formation of polymeric pigments over time is higher in the control than AG20. Several factors, such as the anthocyanins–tannins ratio, the presence of acetaldehyde and other reactive carbonyls, and phenolic acids [63,64], may determine a different reactivity of monomeric anthocyanins in the formation of polymeric pigments over time. The lower content of polymeric pigments in AG20 (Figure 3B) could be explained considering the degradation of ethyl-linked flavanols over time [65]. It is well known that ethyl-bridged compounds are rapidly formed in oxidative conditions [66,67], but they also rapidly broken down. Zhang et al. [68] showed that different classes of anthocyanin derivatives had different distribution patterns as a function of red wine age, and pyranoanthocyanins (except for vitisin B) were deemed to be more stable than the flavanol-related anthocyanin derivatives. In general, pinotins were viewed as the most stable pigments, followed by Flavanyl-pyranoanthocyanins and vitisin A. In a more recent paper, Zhang et al. [69] also showed that oxidation facilitated the formation of pyranoanthocyanins. Since a higher concentration of copper (II) accelerated all reactions with oxygen [37], this hypothesis can be considered as a possible explanation for the lower levels of polymeric pigments (with a molecular size ranging from dimers to oligomers) in AG20. Also, Ćurko et al. [43] observed that in a Plavac mali red wine that was micro-oxygenated and had copper (II) and iron added, the concentrations of tannins and flavan-3-ols were lower than in not treated wines, indicating that the addition of iron and copper (II) can slightly accelerate a decrease in these compounds during aging probably due to the formation of ethyl-bridged compounds and their further reactions [68,70].

**Figure 4:**
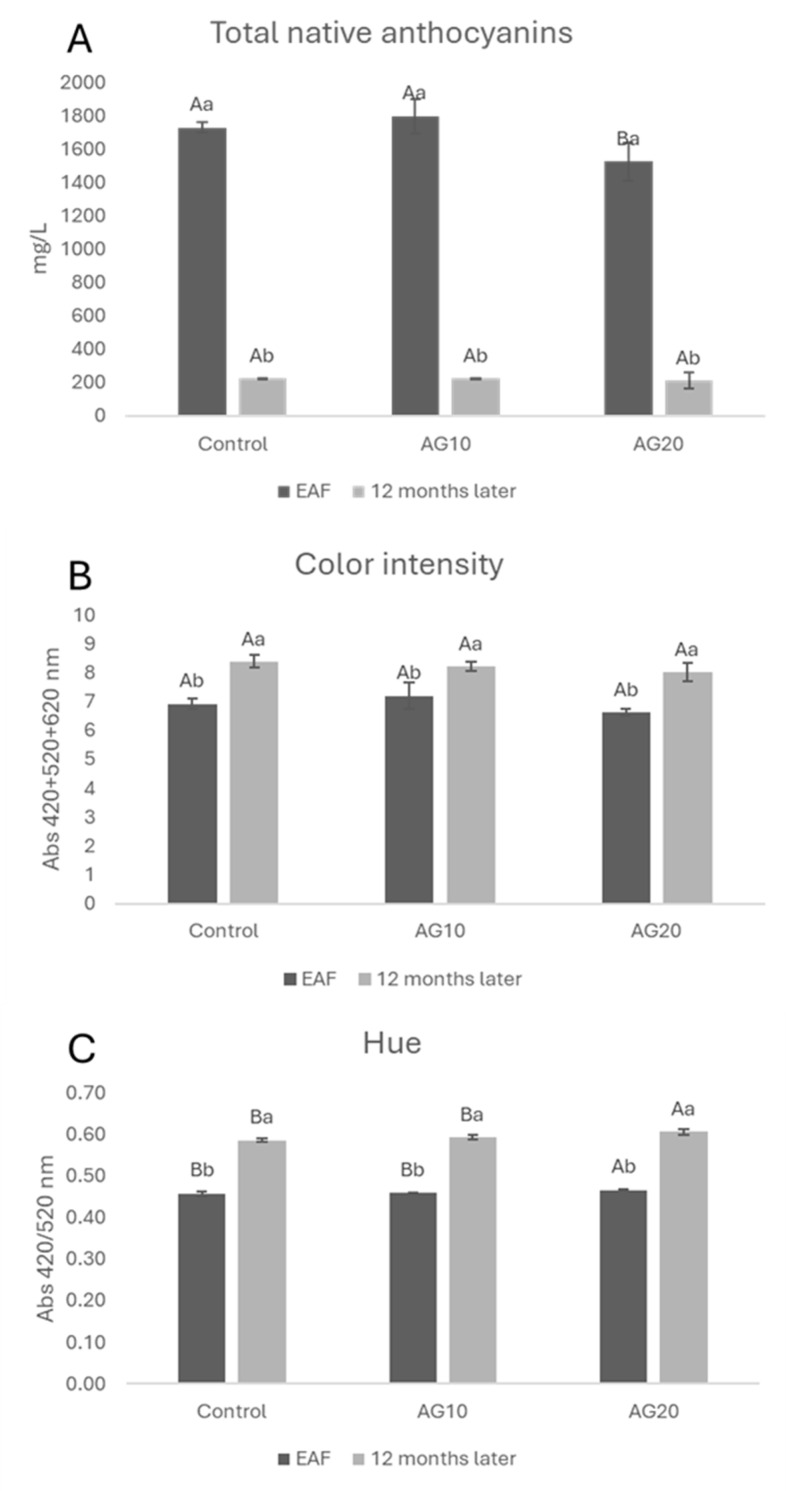
Total native anthocyanins (**A**), color intensity (**B**), hue (**C**) at the end of alcoholic fermentation (EAF) and after 12 months of aging. Data are presented as the mean ± standard deviation (SD) across four replicates. Letters (A, B) indicate statistically significant differences between treated wines at the same time point. Letters (a, b) indicate significant differences for a single treated group over different time points (*p* < 0.05).

During the 12 months of aging, all wines exhibited a decrease in monomeric anthocyanins (Figure 4A), which was accompanied by a rise in polymeric pigments (Figure 3B). These changes resulted in a shift in the wines’ color characteristics in terms of CI and hue (Figure 4B,C). More specifically, all wines showed an increase in both color intensity and hue compared to the EAF stage.

Interestingly, the total amount of native anthocyanins turned out to be basically the same in all the samples after 12 months of aging (Figure 4A).

Among the wines, AG20 displayed statistically significant changes in hue and in the sum of polymeric pigments with respect to both the control and AG10. This might be explained by taking into account the higher degree of anthocyanin degradation in AG20 with the consequent formation of orange pigments (Figure 4C) [68]. This hypothesis is supported by the fact that in AG20, after 12 months, the sum of polymeric red pigments is (Figure 3B) lower for AG20 compared with the control and AG10.

**Figure 5:**
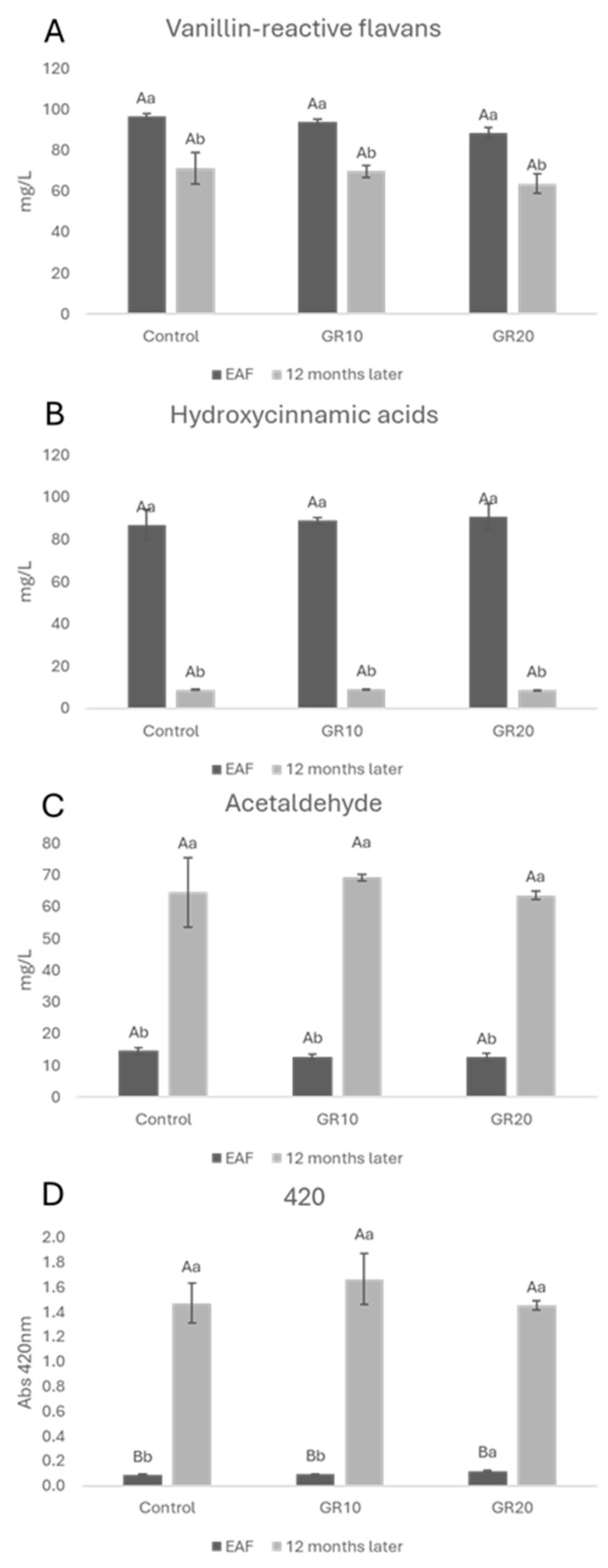
Vanillin-reactive flavans (**A**), hydroxycinnamic acids (**B**), acetaldehyde (**C**), and absorbance 420 nm (**D**) at the end of alcoholic fermentation (EAF) and after 12 months of aging. Data are presented as the mean ± standard deviation (SD) across four replicates. Letters (A, B) indicate statistically significant differences between treated wines at the same time point. Letters (a, b) indicate significant differences for a single treated group over different time points (*p* < 0.05).

Data on the vanillin-reactive flavans (VRFs), hydroxycinnamic acids (HCAs), acetaldehyde, and absorbance at 420 nm (Abs420 nm) of white wine at the EAF and after 12 months of aging are shown in Figure 5.

Regarding HCAs, a loss in all samples after 12 months of aging was detected with respect to the EAF. Acetaldehyde, which is the primary wine oxidation product, increased in all samples regardless of the added copper (II) amount. Such a strong increase can be related to the low amount of free sulfur dioxide in wines (around 4 mg/L in all the samples) that cannot limit the production of acetaldehyde and other oxidation products by the Fenton reaction [71]. This parameter could be associated with the increase in the absorbance at 420 nm (Figure 5D) that measures the amount of brown oxidated pigments in musts and wines. It is likely that in the absence of SO_2_, the formation of glyoxylic acid from tartaric acid and the consequent formation of xanthilium salts [72] were also favored. No effect of copper (II) on the absorbance at 420 nm was detected over time. This is in agreement with the results described by George et al. [55] that showed that the 440 nm absorbance intensity of the copper (II) (II)-containing samples was in the same order as the samples without copper (II) (II), thus highlighting that iron, more than copper (II), is efficient in the production of xanthylium cation pigments in wine-like solutions containing tartaric acid and (+)-catechin. VRFs as well as HCAs decreased during aging (Figure 5A,B). These results are in agreement with a previous study in which, during bottle storage, a decrease in the concentration of catechin and *trans*-caftaric acid was observed [73]. The authors also found that the browning index was more pronounced as a direct function of catechin and trans-caftaric acid degradation. The observed decrease in flavanols can be attributed to their reactivity [74]. No significative differences were detected in all of the samples after 12 months of aging with respect to the control, thus confirming the obtained data on the browning index (Figure 5C).

## 3. Materials and Methods

### 3.1. Biological Materials

Aglianico and Greco di Tufo grapes were furnished by the Quintodecimo winery, which is located in Mirabella Eclano (Southern Italy) in 2020.

The S. cerevisiae strains used in this study were as follows: Zymaflore X5 for Greco di Tufo and Zymaflore FX10 for Aglianico (Laffort Oenologie, Bordeaux, France), along with Thiazote^®^PH and Lafazym^®^CL obtained from the same supplier. Ringer’s solution, WL-nutrient agar (Oxoid, Basingstoke, UK), and Lysine medium (Oxoid) were used for culturing microorganisms. All solvents were of HPLC grade or higher. Glacial acetic acid, hydrochloric acid, methanol, acetonitrile, ethanol, sodium dodecyl sulfate (SDS), triethanolamine, iron chloride, vanillin, tartaric acid, formic acid, sulfuric acid, 2,4-dinitrophenylhydrazine, sodium hydroxide, copper (II) sulfate pentahydrate (CuSO_4_ • 5H_2_O), bovine serum albumin (BSA), malvidin-3-O-monoglucoside, and metabisulfite were purchased from J.T. Baker (Levanchimica, Bari, Italy). Water was purified using a Milli-Q purification system (MilliporeSigma, Burlington, MA, USA).

### 3.2. Fermentation Experiments

The microvinification process was performed at the Grape and Wine Science Division of University of Naples ‘Federico II’.

Red wines: Aglianico red grapes, characterized by a content of soluble solids of 23.4°Brix, were harvested in 2020. Before destemming and crushing, Aglianico grapes were washed and dried in order to eliminate all the copper (II) derived from the cultivation practices. The solid part was separated by a steel sieve from the liquid in order to obtain six samples (3 × 2 replicas) with the same skin–must ratio and different amounts of copper (II). The experimental samples were obtained in 10 L tanks composed of 6.5 L of must and 1.6 Kg of skins. Before alcoholic fermentation, a different amount of copper (II) was added to obtain three different experimental samples: control (no copper (II)), AG10 (must added with 10 mg/Kg of copper (II)), and AG20 (must added with 20 mg/Kg of copper (II)).

Fermentations took place at 20 ± 1 °C after yeast inoculation (20 g/hL of FX10 Zymaflore), the cap was immersed twice a day, and the fermentation was monitored by the evaluation of the solid soluble content.

White grapes: Greco di Tufo grapes were washed, dried, destemmed, and crushed as reported above for the red Aglianico grapes. They were characterized by a content of soluble solid of 21.7° Brix. Before decantation, 1 g/hL of pectolytic enzymes (Lafazym^®^ CL)was added. A limpid must (9.36 ± 0.17 NTU) was then obtained by static decantation in a stainless steel tank at 8 °C for 24 h. Once obtained, and before alcoholic fermentation, a different amount of copper (II) was added to obtain six samples (3 × 2 replicas) as follows: control (no copper (II)), GR10 (must added with 10 mg/L of copper (II)), and GR20 (must added with 20 mg/L of copper (II)).

Fermentations took place at 18 ± 1 °C after yeast inoculation (20 g/hL of X5 Zymaflore), and the fermentation was monitored by the evaluation of the solid soluble content.

At the middle of the alcoholic fermentation, since white grape must is relatively poor in nitrogen-containing compounds (below 200 mg/L), which are essential for yeast growth and fermentation, in all of the samples, 30 g/hL of Thiazote^®^ (formulated with ammonium phosphate and thiamine) was added.

After the fermentation process, both wines were decanted, cold-stabilized at −6 °C, and added with potassium metabisulphite according to standard winemaking protocols before bottled aging. Experimental wine was analyzed 6 days after the end of alcoholic fermentation (EAF) and after 1 year of aging (12 months later). The wines were stored in a wine cellar for aging, in bottles protected from sunlight at 12 °C. All experiments were carried out in duplicate, and two analytical replicates were performed.

### 3.3. Fermentation Experiments

Musts (after yeast inoculation) at zero time (dd0) and wine samples collected during fermentation (dd2, dd5, dd9, dd13, dd20, and dd23 days (dd) for AG; dd2, dd5, dd9, dd13, dd16, dd26, and 34 days for GR) were serially diluted in quarter-strength Ringer’s solution and spread-plated (in duplicate) on WL-nutrient agar supplemented with 100 mg/L of chloramphenicol and on lysine medium for total yeast and non-Saccharomyces counts, respectively. Plates were incubated at 30 °C for 5 days.

### 3.4. Base Parameter Analyses

The analysis of fundamental wine parameters followed established international protocols outlined in the OIV (International Organisation of Vine and Wine) Compendium (2024 edition) for wine and must analysis. These parameters included the following: alcohol content (% *v*/*v*), residual sugars (g/L), total acidity (g/L tartaric acid equivalent), pH, and volatile acidity (g/L acetic acid equivalent). The detailed results for these measurements can be found in Supplementary Tables S1 and S2.

### 3.5. Wine Color and Spectrophotometric Analyses

The color analysis of wine was conducted employing a fusion of the CIELAB color space and spectrophotometric techniques. The CIELAB model is a three-dimensional framework that quantifies color through three attributes: L* (lightness, ranging from 0 for black to 100 for white), a* (representing red–green chromaticity, with positive values indicating redness and negative values indicating greenness), and b* (representing yellow–blue chromaticity, with positive values indicating yellowness and negative values indicating blueness). The measurements of L*, a*, and b* were conducted using NomaSense Color P100, a solution provided by Vinventions, France. Color discrepancies (ΔE⁄ab) were computed as the Euclidean distance within the 3D space defined by L*, a*, and b*. Wine color intensity (CI), determined by the cumulative absorbances at 420 nm (yellow), 520 nm (red), and 620 nm (blue), along with hue (420/520 absorbance), were assessed according to the Glories method [75].

The quantification of hydroxycinnamic acids (HCAs) in wine samples followed a method outlined by Vlahou et al. [76]. In brief, 1 mL of wine sample was diluted at a ratio of 1:10 with 0.1 N H_2_SO_4_. The absorbance of the diluted solution was measured at 325 nm using a spectrophotometer. The concentration of HCAs was expressed as mg/L of p-coumaric acid equivalents utilizing a standard curve prepared with p-coumaric acid. The linear range spanned 2–500 mg/L, with an intercept of 0.024 and a slope of 0.0886.

The determination of vanillin-reactive flavanols (VRFs) followed the procedure outlined by Gambuti et al. [67]. Briefly, a test tube was prepared by diluting wine at a ratio of 1:10 with pure methanol. Two microcentrifuge tubes were then employed. In the first microcentrifuge tube, 125 μL of diluted wine was mixed with 750 μL of a vanillin solution (4% in methanol), followed by the addition of 375 μL of concentrated hydrochloric acid. After 15 min of incubation in cold water and subsequent incubation at room temperature for 15 min, absorbance was measured at 500 nm. The second tube followed the same procedure, substituting the vanillin solution with pure methanol to serve as the blank. The concentration was calculated in (+)-catechin (mg/L) using a calibration curve, with a linear range of 2–250 mg/L. The intercept and slope varied as follows: 0.02 < ΔE < 0.05 (slope 277.26, intercept −3.58); 0.05 < Δ < 0.18 (slope 250, intercept −3.25); and 0.18 < Δ < 0.83 (slope 314.23, intercept −12.91).

Total anthocyanins, BSA tannins, total phenolics, small polymeric colors (SPPs), and large polymeric pigments (LPPs) were determined utilizing the assay outlined by Harbertson et al. [48]. This assay combines protein precipitation using bovine serum albumin (BSA) and bisulfite bleaching to discern two types of polymeric colors in wines: LPPs, which precipitate with proteins, and SPPs, which do not. The determination of HCAs and chromatic characteristics was conducted using a 7305 spectrophotometer (Jenway Bibby Scientific Limited, Stone, UK) with 10 mm plastic cuvettes. All analyses were conducted with two experimental replicates and two analytical replicates.

### 3.6. Determination of Copper (II) Content

Total copper (II) analyses were carried out after homogenizing the mass as reported in the multielement analysis using ICP-MS OIV-MA-AS323-07 [77] by the Laboratorio Ambientale Gamma (Avellino, Italy). The analysis time zero corresponds to four hours from the sampling.

### 3.7. Determination of Monomeric and Polymeric Phenolics

The separation and quantification of monomers and polymeric phenols were conducted following the procedure outlined by Waterhouse et al. [78]. The HPLC setup comprised a Shimadzu LC10 ADVP HPLC system (Shimadzu, Italy, Milan) equipped with an SCL-10AVP system controller, two LC-10ADVP pumps, an SPD-M 10 AVP detector, a Shimadzu CTO-10ASvp column oven, a Shimadzu Sil-20AHT injection autosampler, and a complete Rheodyne model 7725 injection system (Rheodyne, Cotati, CA, USA).

A reversed-phase polystyrene–divinylbenzene column (Agilent PLRP-S 100-Å 4.6 × 150 mm, 3 μm) protected with a cartridge containing the same packing material (PLRP-S, 5 × 3 mm) and maintained at 35 °C served as the stationary phase. Monomeric anthocyanins and polymeric anthocyanins were identified by monitoring absorbance signals at 520 nm. All samples were filtered through 0.20 μm PTFE membrane filters (DWK Life Sciences, Wheaton, Milville, NJ 08332 USA) into dark glass vials and promptly injected into the HPLC system.

The eluents for the HPLC comprised Detergent A: 1.5% *v*/*v ortho*-phosphoric acid (EMP Chemicals, Gibbstown, NJ, USA) and Detergent B: 80% acetonitrile (HPLC grade, Honeywell, Muskegon, MI, USA) with 20% of solvent A. A gradient was applied as follows: 0-time conditions: B 6%; 73 min: B 31%; 78 min: B 62%, remaining constant until 86 min; and 90 min: B 6%. This solvent mixture at 0 time was followed by a 15 min equilibrium period before the injection of the next sample.

The injection of 50 μL of wine or calibration standards into the column was performed. The mobile phase flow rate was set at 1.0 mL/min. A calibration curve was established by injecting 5 sample solutions (in triplicate) with increasing concentrations of malvidin-3-*O*-monoglucoside (Extrasynthese, Lyon, France). All analyses were conducted with two experimental replicates and two analytical replicates.

### 3.8. Determination of Acetaldehyde

Total acetaldehyde was quantified following the procedure outlined by Han et al. [79]. In this method, aliquots of the wine sample (100 μL) were transferred into a vial, to which 20 μL of a 1.120 mg/L SO_2_ solution, prepared from a stock mixture of K_2_S_2_O_5_ (2 g/L), was added. Subsequently, 2 μL of 25% sulfuric acid (96% Carlo Erba, Milan, Italy) and 140 μL of 2 g/L dinitrophenylhydrazine reagent (Merck KGaA, Darmstadt, Germany) were sequentially introduced. After thorough mixing, the vial was allowed to react for 15 min at 65 °C in a laboratory oven before cooling to room temperature.

The analysis was conducted using HPLC, employing the same apparatus utilized for the chromatographic analyses of anthocyanins. A Waters Spherisorb column (250 × 4.6 mm, 4 mm particle diameter) facilitated the separation. The chromatographic conditions were as follows: a sample injection volume of 50 µL, flow rate of 0.75 mL/min, column temperature set at 35 °C, and mobile phase eluents consisting of (A) 0.5% formic acid (Sigma Aldrich ≥ 95%, Saint Louis, MO, USA) in Milli-Q water (Sigma Aldrich) and (B) acetonitrile (Sigma Aldrich ≥ 99.9%).

The gradient elution protocol was initiated at 35% B, reaching 60% B by 8 min, then progressing to 90% B by 13 min, and finally achieving 95% B by 15 min, where it was held for 2 min. Subsequently, a return to 35% B was performed over 4 min, resulting in a total run time of 21 min. Eluted peaks were compared with derivatized acetaldehyde standard (≥99.5%, Sigma Chemistry, USA) obtained after reaction with dinitrophenylhydrazine reagent (Sigma Chemistry, USA).

Calibration curves were generated by analyzing 5 solutions (each in triplicate) containing standards at varying concentrations. The linear range spanned 10–120 mg/L, with a correlation coefficient R^2^ > 0.976. All analyses were conducted with two experimental replicates and two analytical replicates.

### 3.9. Statistical Analysis

Statistical analysis was performed using IBM SPSS Statistics (version number, 29.0.1.0). An analysis of variance (one-way ANOVA) was used to assess differences between groups when data satisfied the assumptions of normality and homogeneity of variance. When these assumptions were violated, the non-parametric Kruskal–Wallis test was employed. All statistical tests were considered significant at *p* < 0.05. All analyses were performed in quadruplicate (2 experimental replicates × 2 analytical replicates). Mean values and standard deviations were determined.

## 4. Conclusions

The present study evaluated the effect of copper (II) on the evolution of acetaldehyde and phenolic substances during fermentation and after 12 months of aging of white and red wines. Our results demonstrated that the initial amount of copper (II) in grapes is of particular importance because at the EAF of white grapes added with 20 mg/L of copper (II), a concentration higher than the legal limit of 1.0 mg/L in wines was detected. For these samples, values below the threshold limit were observed only after 12 months of aging. However, it is difficult to have such a high concentration of copper (II) in musts when complying with legal limits and winemaking good practices. Conversely, during the production of red wines, pomaces probably induce mineral adsorption phenomena by subtracting copper (II) from the medium; hence, concentrations of copper (II) below the legal limit are already observed at the end of fermentation.

Although no differences in copper (II) content among red wines were detected, a significant effect on the phenolic compounds and chromatic characteristics of wines just after the EAF and even after one year of aging was observed. A slight loss in terms of total phenols, vanillin-reactive flavans, and BSA-reactive tannins was observed in AG20 (Aglianico 20 mg/kg). At the EAF, red wine samples treated with a higher amount of copper (II) showed a lower content of total anthocyanins, vanillin-reactive flavans, and BSA-reactive tannins and a lower color intensity and a higher hue. After 12 months of aging, such treated wines were still different by hue and polymeric pigment contents.

These data could ultimately help winemakers, concerned about issues related to ever-increasing copper (II)-based treatments, to properly manage grapes treated with copper (II) before winemaking. Finally, this study underlined the necessity of monitoring copper (II) on musts especially when copper (II) treatments are applied in vineyards.

## Figures and Tables

**Table 1:** Evolution of copper (II) content during alcoholic fermentation in red Aglianico wine.

Aglianico Red Grape					
	Copper (II) (mg/L)				
Time zero	Control	5.35	±	0.64	C a
	AG10	12.70	±	0.20	B a
	AG20	18.22	±	2.50	A a
day 1	Control	5.50	±	0.19	C a
	AG10	10.67	±	0.98	B a
	AG20	15.50	±	1.65	A ab
day 5	Control	2.26	±	0.23	A b
	AG10	5.99	±	0.25	A b
	AG20	14.52	±	7.98	A ab
day 8	Control	1.90	±	0.14	A b
	AG10	7.06	±	1.66	A b
	AG20	3.61	±	4.63	A ab
EAF	Control	0.70	±	0.28	A c
	AG10	0.57	±	0.27	A c
	AG20	0.82	±	0.40	A b

Different letters indicate a statistically significant difference between treated wines: within the same time point: Letters (A, B, C) show significant differences between treated groups (e.g., AG10 vs. AG20) at a single point in time (e.g., day 1). Over time: Letters (a, b, c) show significant differences for a single treated group (e.g., AG10) across different time points (e.g., day 1 vs. day 5). The data are expressed as means ± standard deviation, (*p* < 0.05) over four replications.

**Table 2:** Evolution of copper (II) content during alcoholic fermentation in white Greco di Tufo wine.

Greco di Tufo					
	Copper (II) (mg/L)				
Time zero	Control	3.94	±	0.27	C a
	GR10	9.73	±	0.33	B b
	GR20	16.22	±	0.05	A ab
day 1	Control	4.38	±	0.04	C a
	GR10	11.51	±	0.23	B a
	GR20	19.07	±	0.12	A a
day 4	Control	4.37	±	0.49	C a
	GR10	11.37	±	0.35	B a
	GR20	18.01	±	0.34	A ab
day 8	Control	3.22	±	0.18	C ab
	GR10	11.72	±	0.23	B a
	GR20	18.99	±	1.51	A a
day 12	Control	3.11	±	0.89	C ab
	GR10	9.81	±	0.37	B b
	GR20	16.40	±	0.35	A ab
day 15	Control	3.39	±	0.33	C a
	GR10	9.30	±	0.45	B b
	GR20	15.97	±	1.19	A b
day 25	Control	1.46	±	0.31	B bc
	GR10	2.56	±	0.39	AB c
	GR20	3.72	±	0.61	A c
EAF	Control	0.71	±	0.04	C c
	GR10	1.07	±	0.08	B d
	GR20	1.66	±	0.04	A c

Different letters indicate a statistically significant difference between treated wines: within the same time point: Letters (A, B, C) show significant differences between treated groups (e.g., GR10 vs. GR20) at a single point in time (e.g., day 1). Over time: Letters (a, b, c, d) show significant differences for a single treated group (e.g., GR10) across different time points (e.g., day 1 vs. day 5). The data are expressed as means ± standard deviation, (*p* < 0.05) over four replications.

**Table 3:** Evolution of color intensity, hue, total native anthocyanins, and polymeric pigments in Aglianico red wines.

		Color Intensity ^†^				Hue ^‡^				Total Native Anthocyanins ^§^				Polymeric Pigments ^¶^			
Time zero	Control	2.05	±	0.17	B e	0.82	±	0.02	A a	913.16	±	89.89	A d	14.17	±	11.42	A e
	AG10	2.76	±	0.01	A e	0.82	±	0.01	A a	709.69	±	69.22	B d	18.20	±	1.89	A e
	AG20	2.73	±	0.23	A d	0.82	±	0.04	A a	701.20	±	28.27	B d	16.46	±	3.75	A e
day 1	Control	3.93	±	0.00	B d	0.44	±	0.00	C c	1571.28	±	37.24	A c	29.32	±	6.99	C d
	AG10	4.39	±	0.27	A d	0.47	±	0.01	B b	1588.89	±	270.14	A c	49.94	±	9.23	BC d
	AG20	3.97	±	0.16	B c	0.48	±	0.00	A b	1435.28	±	65.38	A c	63.83	±	13.27	A d
day 5	Control	4.96	±	0.02	A c	0.40	±	0.01	C e	2385.45	±	114.52	A a	69.89	±	5.46	B c
	AG10	5.07	±	0.13	A c	0.41	±	0.00	B e	2144.04	±	39.96	B b	78.17	±	2.17	B c
	AG20	5.08	±	0.23	A b	0.43	±	0.00	A d	2116.72	±	82.40	B b	103.08	±	10.92	A c
day 8	Control	6.26	±	0.15	A b	0.41	±	0.00	C d	2495.33	±	133.27	B a	139.70	±	11.46	B b
	AG10	6.43	±	0.29	A b	0.42	±	0.00	B d	2581.75	±	232.46	A a	159.39	±	17.68	AB b
	AG20	5.46	±	0.08	B b	0.53	±	0.06	A b	2458.77	±	49.06	AB a	175.36	±	3.48	A b
EAF	Control	6.92	±	0.17	A a	0.46	±	0.01	B b	1728.20	±	33.28	A b	253.56	±	11.84	B a
	AG10	7.19	±	0.46	A a	0.46	±	0.00	B c	1796.57	±	104.26	A c	281.94	±	23.85	AB a
	AG20	6.61	±	0.12	B a	0.47	±	0.00	A c	1524.18	±	115.47	B c	267.41	±	4.12	A a

Data are presented as the mean ± standard deviation (SD) across four replicates. Letters (A, B, C) indicate statistically significant differences between treated wines at the same time point. Letters (a, b, c, d, e) indicate significant differences for a single treated group over different time points (*p* < 0.05). ^†^ CI, color intensity, the sum of 420, 520, 620 nm; ^‡^ hue is the 420 nm/520 nm ratio; ^§^ total native anthocyanin is expressed as mg/L of malvidin-3-glucoside equivalent; ^¶^ polymeric pigments are expressed as mg/L of malvidin-3-O-monoglucoside equivalent.

**Table 4:** Evolution of total phenols, BSA-reactive tannins, and vanillin-reactive flavans in Aglianico red wines.

		Total Phenols ^†^				BSA-Reactive Tannins ^‡^				Vanillin-Reactive Flavans ^§^			
Time zero	Control	814.29	±	58.99	B e	103.19	±	25.58	B e	126.32	±	33.13	A e
	AG10	1038.41	±	28.93	A e	176.69	±	16.08	A e	137.50	±	11.37	A e
	AG20	1052.33	±	29.11	A e	170.81	±	9.37	A e	112.18	±	67.74	A e
day 1	Control	1304.29	±	36.48	A d	225.06	±	9.59	A d	281.25	±	54.56	A d
	AG10	1322.39	±	30.33	A d	240.17	±	11.25	A d	268.75	±	47.50	A d
	AG20	1302.90	±	42.41	A d	237.79	±	15.40	A d	318.13	±	55.17	A d
day 5	Control	1702.42	±	111.12	A c	448.58	±	10.43	A c	684.76	±	30.89	A c
	AG10	1607.76	±	143.22	A c	428.85	±	38.56	AB c	592.06	±	62.08	AB c
	AG20	1641.17	±	45.32	A c	392.90	±	25.61	B c	583.42	±	53.96	B c
day 8	Control	2094.97	±	144.23	A b	569.17	±	41.58	A b	796.31	±	23.86	A b
	AG10	2099.15	±	71.45	A b	570.76	±	9.65	A b	801.81	±	51.85	A b
	AG20	1894.52	±	45.55	B b	526.22	±	22.50	A b	716.18	±	28.69	B b
day 19	Control	2828.58	±	74.98	AB a	640.68	±	36.59	A a	1533.18	±	29.92	A a
	AG10	2881.48	±	18.47	A a	690.80	±	27.32	A a	1599.17	±	46.40	A a
	AG20	2763.16	±	95.62	B a	575.85	±	20.41	B a	1420.84	±	18.39	B a

Data are presented as the mean ± standard deviation (SD) across four replicates. Letters (A, B) indicate statistically significant differences between treated wines at the same time point. Letters (a, b, c, d, e) indicate significant differences for a single treated group over different time points (*p* < 0.05). ^†^ Total phenols (mg/L); ^‡^ BSA-reactive tannins expressed as mg/L catechin equivalent; ^§^ vanillin-reactive flavans (mg/L).

**Table 5:** Hydroxycinnamic acids, Abs 420 nm, and total phenols in white wines at the EAF.

		HCAs ^†^				Abs 420 nm ^‡^				Total Phenols ^§^			
Time zero	Control	124.13	±	0.52	A a	0.16	±	0.01	A a	405.03	±	74.48	A a
	AG10	124.13	±	0.52	A a	0.16	±	0.01	A a	405.03	±	74.48	A a
	AG20	124.13	±	0.52	A a	0.16	±	0.01	A a	405.03	±	74.48	A a
EAF	Control	86.88	±	7.30	A b	0.09	±	0.00	B b	420.34	±	39.76	A a
	AG10	88.94	±	1.14	A b	0.09	±	0.00	B b	417.56	±	46.81	A a
	AG20	90.81	±	5.96	A b	0.12	±	0.01	A b	396.68	±	59.43	A a

Data are presented as the mean ± standard deviation (SD) across four replicates. Letters (A, B) indicate statistically significant differences between treated wines at the same time point. Letters (a, b) indicate significant differences for a single treated group over different time points (*p* < 0.05). ^†^ (HCAs) hydroxycinnamic acids are expressed as mg/L of p-coumaric acid; ^‡^ Abs 420 nm is the absorbance at 420 nm; ^§^ total phenols are expressed as mg/L.

## Data Availability

The raw data supporting the conclusions of this article will be made available by the authors on request.

## Supplementary Materials

The following supporting information can be downloaded at: https://www.mdpi.com/article/10.3390/molecules29122907/s1, Table S1: Base parameters of red Aglianico must and white Greco di Tufo must used for the experiment; Table S2: Base parameters of red Aglianico wines and white Greco di Tufo wines just after the end of the alcoholic fermentation (EAF); Table S3: Base parameters of red Aglianico wines and white Greco di Tufo wines after 12 months of aging; Figure S1: Evolution of soluble solids (g/100 g must) (A) Aglianico red grape, (B) Greco di Tufo white grape during the alcoholic fermentations; Figure S2: Monitoring of total yeast (TY) and non-*Saccharomyces* (NS) during AG fermentations without (CN) and with Cu (II) (10 mg/L and 20 mg/L); Figure S3: Monitoring of total yeast (TY) and non-*Saccharomyces* (NS) during GR fermentations without (CN) and with Cu (II) (10 mg/L and 20 mg/L); Table S4: Monomeric anthocyanins of red Aglianico wine at different times of the alcoholic fermentation.

## Abbreviations

Brix	Degree Brix (sugar content)
a*	Red–green chromaticity in CIELab color space
AF	Alcoholic fermentation
AG	Aglianico (red) grape variety
b*	Yellow–blue chromaticity in CIELab color space
BSA	Bovine serum albumin
CI	Color intensity
CIELab	Color space system (L*, a*, b*)
DD	Days after yeast inoculation
EAF	End of alcoholic fermentation
FX10	Zymaflore FX10 (yeast strain)
g/hL	Grams per hectoliter (concentration)
GR	Greco di Tufo (white) grape variety
HCAs	Hydroxycinnamic acids
L*	Lightness in CIELab color space
LPPs	Large polymeric pigments
NTU	Nephelometric turbidity unit
OIV	Organisation Internationale de la Vigne et du Vin
SDS	Sodium dodecyl sulfate
SPPs	Small polymeric colors
VRFs	Vanillin-reactive flavanols
WL	Wallerstein Laboratory nutrient agar
X5	Zymaflore X5 (yeast strain)
Δ	Change or difference in a measurement
ΔE⁄ab	Euclidean distance in CIELab color space
