# Supplementary material for: Copper (II) Level in Musts Affects Acetaldehyde Concentration, Phenolic Composition, and Chromatic Characteristics of Red and White Wines
Source: Molecules. 2024 Jun 19;29(12):2907. doi: 10.3390/molecules29122907 (PMC11206618; doi:10.3390/molecules29122907)
Supplement: Supplementary file 1 [file molecules-29-02907-s001.zip › molecules-3043115-supplementary.pdf]

Supplementary material

Copper (II) level in musts affects the acetaldehyde concentrations, phenolic composition and chromatic characteristics of red and white wines

Francesco Errichiello, Luigi Picariello, Martino Forino *, Giuseppe Blaiotta, Ernesto Petruzziello, Luigi Moio and Angelita Gambuti

Department of Agricultural Sciences, Grape and Wine Science Division, University of Naples "Federico II", 83100 Avellino, Italy; francesco.errichiello@unina.it (F.E.); luigi.picariello@unina.it (L.P.); blaiotta@unina.it (G.B.); ernesto.petruzziello@unina.it (E.P.); moio@unina.it (L.M.); angelita.gambuti@unina.it (A.G.)

* Correspondence: forino@unina.it; Tel.: +39-081-2532604

Supplementary Table S1: Base parameters of red Aglianico must and white Greco di Tufo must used for the experiment.

Supplementary Table S2: Base parameters of red Aglianico wines and white Greco di Tufo wines just after the end of the alcoholic fermentation (EAF).

Supplementary Table S3: Base parameters of red Aglianico wines and white Greco di Tufo wines after 12 months of aging.

Supplementary Figure S1: Evolution of soluble solids (g/100 g must) (A) Aglianico red grape, (B) Greco di Tufo white grape during the alcoholic fermentations.

Supplementary Figure S2: Monitoring of total yeast (TY) and non-*Saccharomyces* (NS) during AG fermentations without (CN) and with Cu (II) (10 mg/L and 20 mg/L)

Supplementary figure S3: Monitoring of total yeast (TY) and non-*Saccharomyces* (NS) during GR fermentations without (CN) and with Cu (II) (10 mg/L and 20 mg/L)

Supplementary Table S4: Monomeric anthocyanins of red Aglianico wine at different times of the alcoholic fermentation.

Must base parameters						
	Total acidity (g/L)	pH	Volatile acidity (g/L)	°Brix	Free SO_2 (mg/L)	Total SO_2 (mg/L)
Aglianico	9.05 ± 0.06	3.39 ± 0.01	0.06 ± 0.00	23.35 ± 0.06	7.68 ± 0.00	35.20 ± 2.71
Greco di Tufo	8.30 ± 0.02	3.15 ± 0.01	0.01 ± 0.00	21.70 ± 0.00	6.40 ± 0.00	25.60 ± 0.00

Supplementary Table S1: Base parameters of red Aglianico must and white Greco di Tufo must used for the experiment. Results are reported as mean values ± SD.

Aglianico

	Total acidity (g/L)	pH	Volatile acidity (g/L)	Ethanol (v/v)	Reducing sugar (g/L)	Free SO_2 (mg/L)	Total SO_2 (mg/L)
Control	8.53 ± 0.04 A	3.41 ± 0.02 A	0.5 ± 0.02 B	13.16 ± 0.04 A	1.16 ± 0.18 A	0.00 ± 0.00	34.56 ± 0.00 A
AG 10	8.55 ± 0.10 A	3.39 ± 0.01 AB	0.52 ± 0.01 B	13.2 ± 0.04 A	1.18 ± 0.13 A	0.00 ± 0.00	28.80 ± 0.74 B
AG 20	8.48 ± 0.05 A	3.38 ± 0.01 B	0.56 ± 0.00 A	13.19 ± 0.06 A	1.14 ± 0.16 A	0.00 ± 0.00	26.24 ± 0.74 C

Greco di Tufo

	Total acidity (g/L)	pH	Volatile acidity (g/L)	Ethanol (v/v)	Reducing sugar (g/L)	Free SO_2 (mg/L)	Total SO_2 (mg/L)
Control	7.98 ± 0.04 B	3.21 ± 0.00 B	0.74 ± 0.01 B	12.65 ± 0.04 A	0.93 ± 0.13 A	0.00 ± 0.00	19.84 ± 5.17 A
Gr 10	7.99 ± 0.03 AB	3.22 ± 0.01 AB	0.8 ± 0.02 AB	12.64 ± 0.06 A	0.93 ± 0.15 A	0.00 ± 0.00	17.92 ± 0.00 A
Gr 20	8.06 ± 0.05 A	3.23 ± 0.01 A	0.8 ± 0.05 A	12.61 ± 0.05 A	0.94 ± 0.18 A	0.00 ± 0.00	18.56 ± 0.74 A

Supplementary Table S2: Base parameters of red Aglianico wines and white Greco di Tufo wines just after the end of the alcoholic fermentation. Results are reported as mean values ± SD. Different letters indicate significant differences (ANOVA: Tukey t-test. $p < 0.05$ - SPSS).

Aglianico

	Total acidity (g/L)				pH				Volatile acidity (g/L)				Ethanol (v/v %)				Free SO_2 (mg/l)				Total SO_2 (mg/l)			
Control	7.69	±	0.13	A	3.29	±	0.04	A	0.56	±	0.02	A	13.43	±	0.80	A	13.12	±	0.64	A	41.28	±	2.19	A
AG 10	7.67	±	0.07	A	3.23	±	0.01	B	0.52	±	0.03	A	14.25	±	0.10	A	10.88	±	0.74	B	39.68	±	1.05	A
AG 20	7.56	±	0.04	A	3.22	±	0.01	B	0.54	±	0.02	A	14.09	±	0.14	A	11.84	±	1.23	AB	40.96	±	2.09	A

Greco di Tufo

	Total acidity (g/L)				pH				Volatile acidity (g/L)				Ethanol (v/v %)				Free SO_2 (mg/l)				Total SO_2 (mg/l)			
Control	6.45	±	0.09	A	3.00	±	0.01	B	0.56	±	0.07	A	12.45	±	0.07	A	4.00	±	0.00	A	81.50	±	8.85	A
GR 10	6.42	±	0.03	A	3.03	±	0.01	A	0.61	±	0.02	A	12.35	±	0.01	B	4.50	±	1.00	A	84.50	±	4.12	A
GR 20	6.45	±	0.02	A	3.02	±	0.00	AB	0.58	±	0.07	A	12.27	±	0.11	B	3.50	±	1.00	A	78.50	±	5.00	A

Supplementary Table S3: Base parameters of red Aglianico wines and white Greco di Tufo wines after 12 months of aging. Results are reported as mean values ± SD. Different letters indicate significant differences (ANOVA: Tukey t-test. $p < 0.05$ - SPSS).

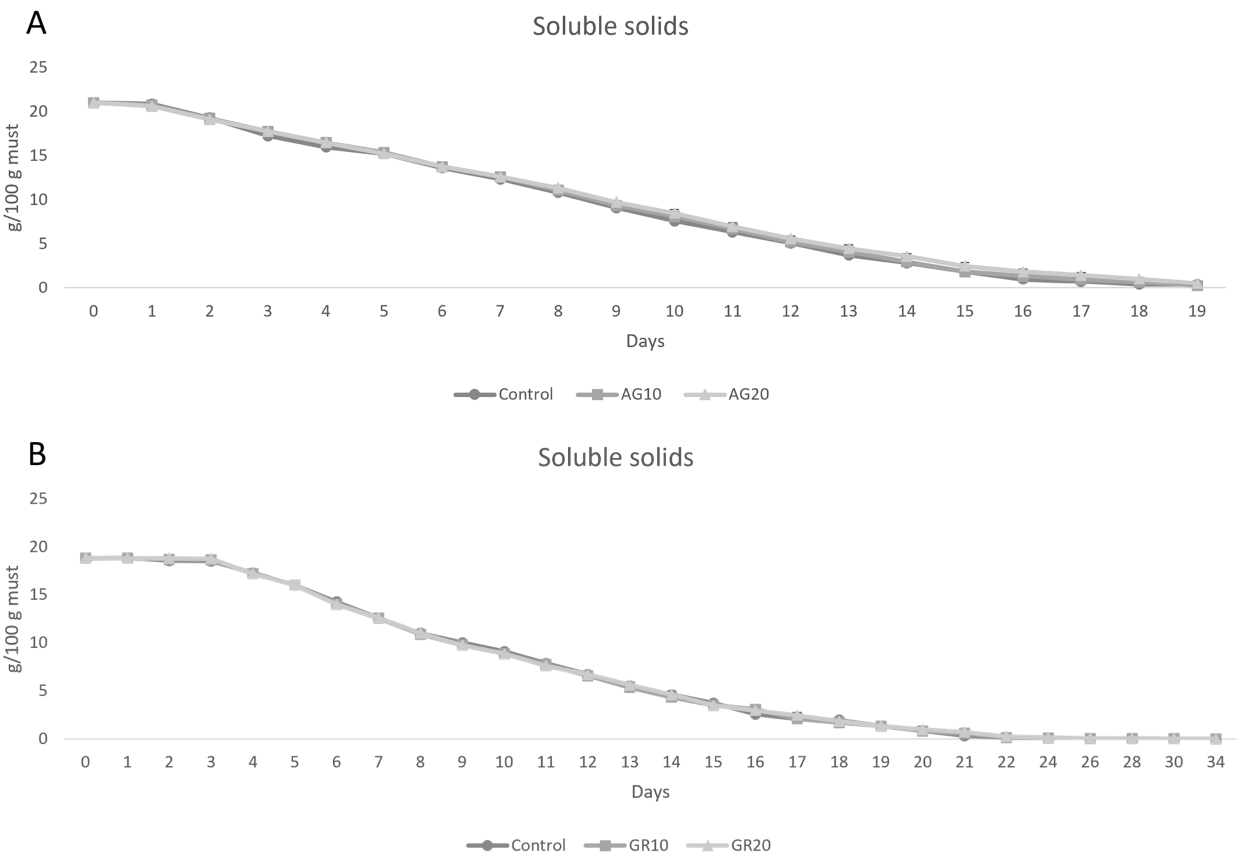

Supplementary Figure S1. Evolution of soluble solids, mainly carbohydrates, expressed as g/100 g must, in Aglianico must (A) and Greco di Tufo must (B) during the alcoholic fermentations. Fermentation times turned out to be different for the two varieties, with that of Greco di Tufo longer than that of Aglianico. No statistically significant differences were detected between treated wines. The data are expressed as means ± standard deviation, ($p < 0.05$) over four replications.

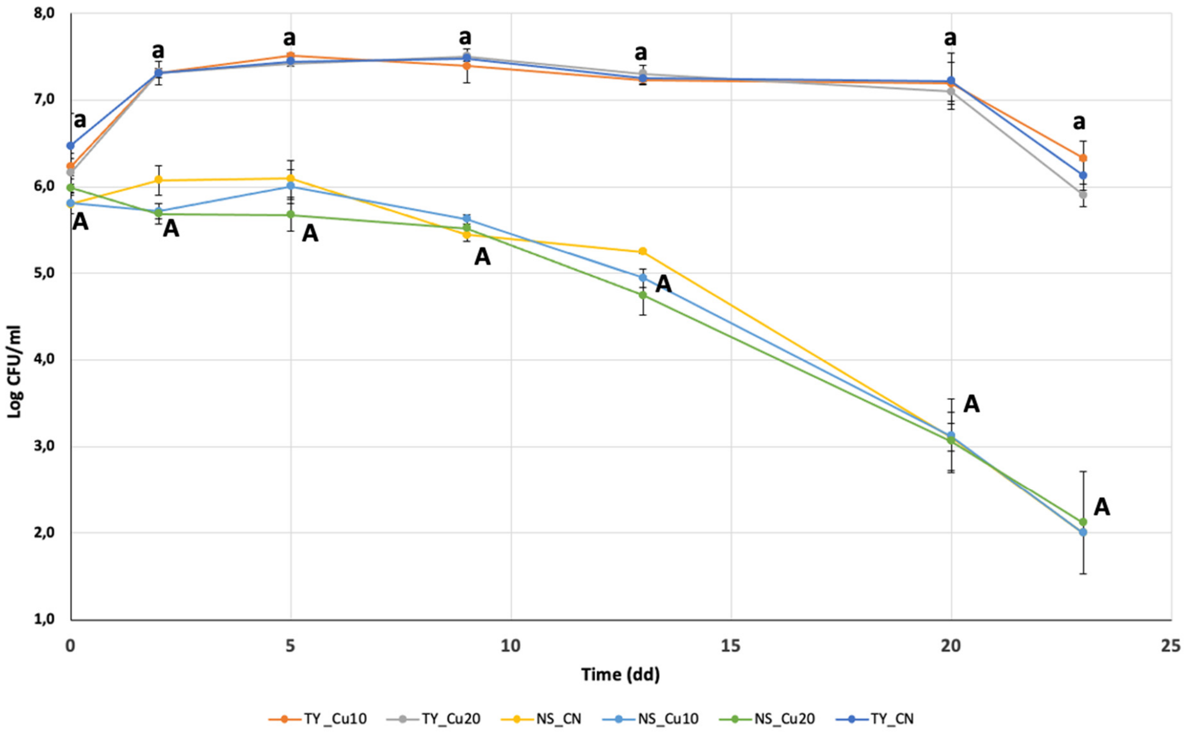

Supplementary Figure S2: Monitoring of total yeast (TY) and non-*Saccharomyces* (NS) during AG fermentations without (CN) and with Cu (II) (10 mg/L and 20 mg/L: Cu10 and Cu20, respectively). Results are reported as mean values ± SD. Different letters indicate significant differences (ANOVA: Tukey t-test. $p < 0.05$ - SPSS). Low-case letters were used for TY and upper-case letters were used for NS.

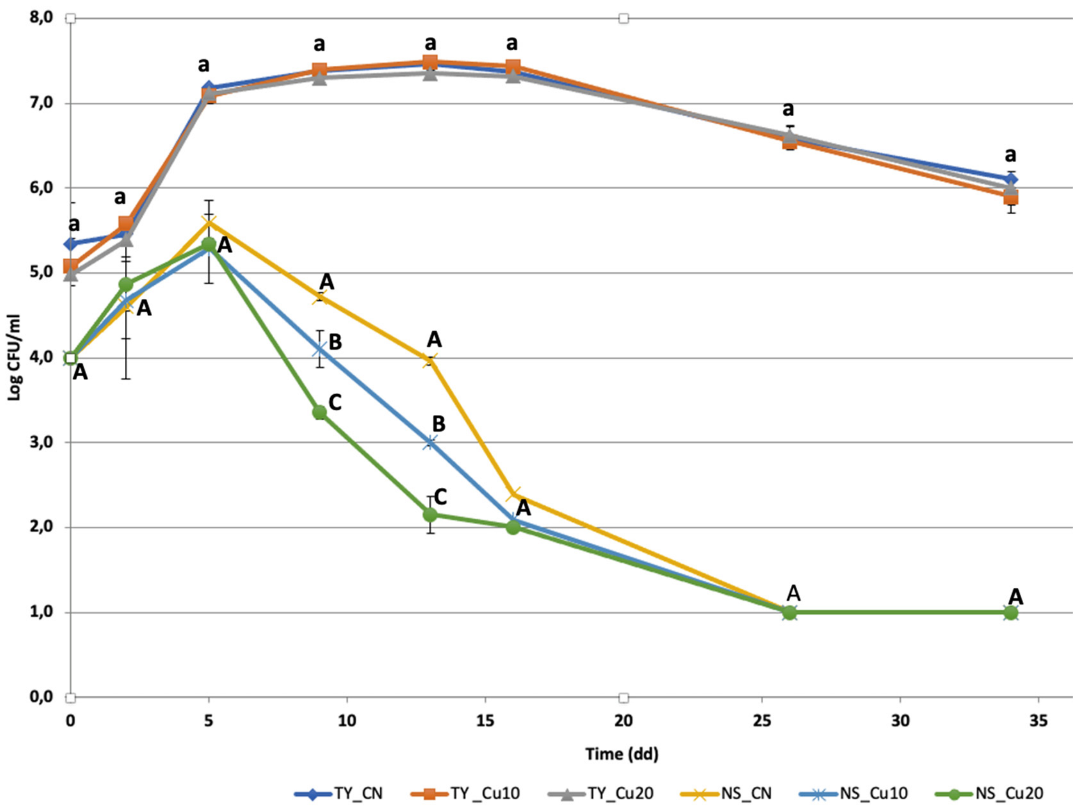

Supplementary Figure S3: Monitoring of total yeast (TY) and non-*Saccharomyces* (NS) during GR fermentations without (CN) and with Cu (II) (10 mg/L and 20 mg/L: Cu10 and Cu20, respectively). Results are reported as mean values ± SD. Different letters indicate significant differences (ANOVA: Tukey t-test. $p < 0.05$ - SPSS). Low-case letters were used for TY and upper-case letters were used for NS.

		Delf-3-*O*-glu	Pet-3-*O*-glu	Peo-3-*O*-glu	Malv-3-*O*-glu	Mal-3-acetyl-glu	Mal-3-Coum-glu
Time zero	Controllo	2.56 ± 1.11 Ae	12.26 ± 0.20 Ab	104.08 ± 6.63 Aab	724.45 ± 57.70 Ad	37.60 ± 8.15 Ad	32.22 ± 21.18 Ae
	AG 10	2.21 ± 0.58 Ac	10.07 ± 2.19 Ad	87.13 ± 8.21 Bb	554.61 ± 53.99 Bd	31.99 ± 2.33 Ad	23.68 ± 3.05 Ad
	AG 20	2.29 ± 0.24 Ae	9.25 ± 1.67 Ae	84.19 ± 3.21 Bb	553.26 ± 23.57 Bd	30.02 ± 2.53 Ae	22.20 ± 1.68 Ad
Day 1	Controllo	76.81 ± 1.96 Ac	74.81 ± 42.11 Ab	107.81 ± 3.79 Aa	1145.16 ± 40.04 Ac	59.78 ± 1.69 Bc	106.91 ± 8.29 Ad
	AG 10	65.35 ± 16.92 Bb	95.21 ± 17.64 Ac	113.16 ± 16.90 Aa	1198.91 ± 166.20 Ac	109.46 ± 22.05 Aa	59.53 ± 16.91 Bc
	AG 20	39.92 ± 1.86 Cd	73.39 ± 2.20 Ad	101.66 ± 3.58 Aa	1063.42 ± 52.61 Ac	54.98 ± 3.02 Bd	101.91 ± 3.83 Ac
Day 5	Controllo	127.59 ± 9.09 Aa	156.21 ± 9.99 Aa	95.48 ± 3.85 Ab	1689.10 ± 75.21 Aa	90.72 ± 4.30 Aa	226.35 ± 12.37 Ab
	AG 10	108.31 ± 2.08 Ba	139.03 ± 2.55 Bb	85.75 ± 5.15 Bb	1532.95 ± 27.70 Bb	82.26 ± 0.95 Bb	195.74 ± 4.74 Bb
	AG 20	95.01 ± 3.26 Cb	130.18 ± 6.96 Bb	87.61 ± 1.65 Bb	1534.08 ± 64.63 Bb	81.65 ± 3.41 Bb	188.19 ± 8.83 Bb
Day 8	Controllo	113.97 ± 4.89 Ab	160.33 ± 8.27 ABa	69.43 ± 9.50 Ac	1798.87 ± 92.43 Aa	97.97 ± 8.26 Aa	254.76 ± 10.48 Aa
	AG 10	115.11 ± 10.97 Aa	173.00 ± 13.96 Aa	70.92 ± 7.53 Ab	1855.01 ± 165.45 Aa	101.62 ± 9.90 Aa	266.08 ± 31.70 Aa
	AG 20	105.24 ± 1.05 Aa	153.17 ± 1.55 Ba	72.52 ± 2.89 Ac	1789.93 ± 45.63 Aa	100.52 ± 1.76 Aa	237.39 ± 6.10 Aa
EAF	Controllo	59.46 ± 2.73 ABd	97.13 ± 0.82 ABb	51.92 ± 2.08 Ad	1262.46 ± 19.31 Ab	66.96 ± 3.11 Ab	190.28 ± 10.33 ABc
	AG 10	64.16 ± 8.21 Ab	110.64 ± 11.31 Ac	53.95 ± 3.23 Ac	1287.89 ± 62.09 Ac	70.11 ± 3.49 Ac	209.82 ± 18.76 Ab
	AG 20	51.46 ± 2.25 Bc	93.69 ± 8.90 Bc	35.68 ± 15.99 Ad	1112.76 ± 76.02 Bc	64.81 ± 2.31 Ac	165.78 ± 19.76 Bb

Supplementary Table S4: Monomeric anthocyanins of red Aglianico wine at different times of the alcoholic fermentation. Results are reported as mean values ± SD. Different letters indicate a statistically significant difference between treated wines: within the same time point: letters (A, B, C, D) show significant differences among treated groups (e.g., AG10 vs. AG20) at a single point in time (e.g., day 1). At different times: letters (a, b, c, d, e) show significant differences for a single treated group (e.g., AG10) across different time points (e.g., day 1 vs. day 5). The data are expressed as means ± standard deviation, ($p < 0.05$) over four replications. Delf-3-*O*-glu: delphinidin-3-*O*-glucoside; Pet-3-*O*-glu: petunidin-3-*O*-glucoside; Peo-3-*O*-glu: peonidin-3-*O*-glucoside; Malv-3-*O*-glu: malvidin-3-*O*-glucoside; Mal-3-acetyl-glu: malvidin-3-*O*-(6-*O*-acetyl)glucoside; Mal-3-Coum-glu: malvidin-3-*O*-(6-p-coumaroyl)glucoside.
